# Are men well served by family planning programs?

**DOI:** 10.1186/s12978-017-0278-5

**Published:** 2017-01-23

**Authors:** Karen Hardee, Melanie Croce-Galis, Jill Gay

**Affiliations:** 1 Population Council, 4301 Connecticut Ave NW, Ste. 280, Washington, DC 20008 USA; 2 What Works Association, 54 Mills St, Morristown, New Jersey 07960 USA

**Keywords:** Family planning, Men, Male engagement, Contraceptive use, Male contraceptive users

## Abstract

Although the range of contraceptives includes methods for men, namely condoms, vasectomy and withdrawal that men use directly, and the Standard Days Method (SDM) that requires their participation, family planning programming has primarily focused on women. What is known about reaching men as contraceptive users? This paper draws from a review of 47 interventions that reached men and proposes 10 key considerations for strengthening programming for men as contraceptive users. A review of programming shows that men and boys are not particularly well served by programs. Most programs operate from the perspective that women are contraceptive users and that men should support their partners, with insufficient attention to reaching men as contraceptive users in their own right. The notion that family planning is women’s business only is outdated. There is sufficient evidence demonstrating men’s desire for information and services, as well as men’s positive response to existing programming to warrant further programming for men as FP users. The key considerations focus on getting information and services where men and boys need it; addressing gender norms that affect men’s attitudes and use while respecting women’s autonomy; reaching adolescent boys; including men as users in policies and guidelines; scaling up successful programming; filling gaps with implementation research and monitoring & evaluation; and creating more contraceptive options for men.

## Plain English summary

Although the range of contraceptives includes methods for men, namely condoms, vasectomy and withdrawal that men use directly, and the Standard Days Method (SDM) that requires their participation, family planning programming has primarily focused on women. What is known about reaching men as contraceptive users? This paper draws from a review of 47 interventions that reached men that are grouped under five strategies: 1) Clinic Provision of Information and Services for Men; 2) Outreach with Male Motivators and Peer Educators/Mentors; 3) Community Engagement; 4) Communications Programs; and 5) Comprehensive Sexuality Education. Based on analysis of these interventions and interviews with experts working on men and family planning, this paper proposes 10 key considerations for strengthening programming for men as contraceptive users. A review of programming shows that men and boys are not particularly well served by programs. Most programs operate from the perspective that women are contraceptive users and that men should support their partners, with insufficient attention to reaching men as contraceptive users in their own right. The notion that family planning is women’s business only is outdated. There is sufficient evidence demonstrating men’s desire for information and services, as well as men’s positive response to existing programming to warrant further programming for men as FP users. The 10 key considerations include: Provide information and services to men and boys where and when they need it; Address gender norms that affect men’s use of contraceptive methods; Meet men’s needs while respecting women’s autonomy; Improve couple and community communication; Link men’s contraceptive use with their desire to support their families; Teach adolescent boys about pregnancy prevention and healthy sexual relationships; Develop national policies and guidelines that include men as family planning users; Scale up programs for men; Fill the gaps through monitoring, evaluation, and implementation science; and Create more contraceptive options for men.

## Background

Reproduction involves both women and men, and although the range of contraceptives includes methods for men, namely condoms, vasectomy and withdrawal that men use directly, and the Standard Days Method (SDM) that requires their participation, family planning programming has primarily focused on women. FP2020’s goal explicitly identifies reaching an additional 120 million women and girls with family planning. Attention to gender at the 1994 International Conference on Population and Development (ICPD) in Cairo resulted in a renewed call to involve men more actively in reproductive health [1, 2]. The framing of ICPD emphasized men as partners to support the autonomous decisions of women, with less regard for men’s reproductive health and rights [3]. More recent efforts to expand the vision for constructive male engagement in family planning and reproductive health are evolving from encouraging men to be supportive partners of women’s reproductive health decisions to also being change agents in families and communities, as well as meeting thir own reproductive health needs [4].

While engaging men improves reproductive health and gender outcomes [2, 5–7], most evidence relates to men as partners. Less is known about reaching men as clients of family planning services. Use of male and male cooperation methods has remained steady over the past few decades at around one-quarter of contraceptive users worldwide, with current regional and country variation in use of the various methods [8]. This paper reviews current programs and evidence, including those that address gender norms that affect men’s use of contraceptive methods, and proposes key considerations to strengthen programs for men as family planning users. The analysis takes a broad view of programming to include policies that create an enabling environment, services and behavior change interventions to address norms and demand creation.

## Methods

This paper is based on evidence from published and grey literature documentation of interventions that included some focus on men as users of contraception in low- and middle-income countries, augmented with interviews with 36 representatives from institutions involved in relevant programming and research [9]. The four methods of male condom, vasectomy, withdrawal and the Standard Days Method, or SDM are included in the analysis.

The literature review included a search strategy for articles and reports from 2010 to 2015, and included selected programming from years prior to 2010 if one or more of these male contraceptive methods was included. To identify current programming that has not made its way into the published literature, relevant abstracts from the January 2016 International Conference on Family Planning were also reviewed. From 2,254 de-duplicated references found through PubMed and POPLINE, the majority of the 500 articles, reports and abstracts reviewed focused on men as supporters of women’s contraceptive use; 47 that included some focus on men as users of contraception were included in the analysis. The information in the interviews was used to ensure that all essential programming was covered, including ongoing programming that may not yet be evaluated as well as used to get expert opinions on the state of programming for men as contraceptive users.

## Results

### Programs for men as contraceptive users

Men have been reached as users of contraception through a range of programming that spans creating demand and expanding supply, in addition to addressing the enabling environment. Programming for men generally falls under the following five broad strategies that are designed to increase demand for and improve the supply of contraceptive information and services. The strategies include: 1) Clinic Provision of Information and Services for Men; 2) Outreach with Male Motivators and Peer Educators/Mentors; 3) Community Engagement; 4) Communications Programs; and 5) Comprehensive Sexuality Education. Two of the strategies, namely communication programs and community engagement, include sub-strategies. Table 1 shows the strategies, the number of interventions found under each strategy, the countries of implementation and the categorization of the strategy as proven, promising or emerging based on the strength of evidence for each strategy adapted from the Family Planning High Impact Practices initiative [10]. Evidence for the strategies ranged from randomized control trails to service statistics. The 47 interventions, their outcomes and the strength of evidence are described in more detail in Hardee et al. [9]. The interventions were carried out in 27 countries spanning Africa, Asia, Latin America and the Caribbean, and the Balkans.

**Table 1:** Strategies to address men and family planning users, number of interventions by strategy, and designation of the interventions as proven, promising or emerging based on the strength of evidence on outcomes

Strategy	Number of Inter-ventions	Countries	Range of Evidence	Categorization of Strategy based on Strength of Evidence
Clinic Provision of Information and Services	4	Bangladesh, Tanzania, and Papua New Guinea, India, Ghana, and Rwanda	Service statistics (3); mystery client study (1)	Promising
Outreach with Male Motivators and Peer Educators/Mentors	10	Malawi, Pakistan, India, El Salvador, Guatemala, India, the Philippines, Nigeria, Madagascar, Timor Leste, Ghana, Ecuador, Nicaragua, and Kenya.	Randomized intervention/control studies (3); pre-post intervention studies using non-randomized intervention and control designs (3); post-intervention survey (1); qualitative interviews and/or focus group discussions (3); service statistics (4)	Proven/Emerging
Communications Programming				
Social marketing	3	Pakistan, Cameroon and Senegal	Pre-post intervention survey (2); post-intervention survey (1)	Proven
Mass Media and Social Media	7	Bangladesh, Ghana, Honduras, Guatemala, Pakistan, India, Vietnam, Burkina Faso, Tanzania, and Nicaragua	Pre-post intervention surveys (4); service statistics (3); FGD (2); In-depth interviews (1)	Promising/Emerging
mHealth	4	Nigeria, Mozambique, India, Ghana, Tanzania and Rwanda	Pre-post intervention study (1) and service statistics (3)	Emerging
Hotlines	3	Uganda and the Democratic Republic of Congo	User call statistics (2); Survey of users of a hotline (1)	Emerging
Community Engagement				
Community Dialogue	10	Kenya, Uganda, South Africa, Nigeria, Pakistan, Brazil, and India	Randomized control trial (2); quasi-experimental, with intervention and control groups (4); baseline/endline surveys (3); and post-intervention qualitative evaluation (1)	Strongly promising (note that this intervention is strongly promising because the evidence for it comes primarily from HIV rather than FP interventions
Engaging Religious Leaders	2	Kenya and Pakistan	Longitudinal survey with baseline and endline (1); baseline/endline survey with qualitative interviews (1)	Emerging
Comprehensive Sexuality Education	4	Tanzania, Uganda, the Balkans and Thailand	Pre-post intervention survey with intervention and control groups (1); indepth interviews long-term post intervention (1); qualitative indepth interviews and focus group discussions (1); pre-post intervention quantitative survey and qualitative interviews (1)	Promising

Not all interventions specifically measured use of male methods, nor disaggregated data by sex, however, it is possible to draw some conclusions about the effect of programming on male use of contraception. Interventions sought to improve men’s attitudes towards family planning, their knowledge of specific methods of contraception, and their use of family planning generally or male methods specifically. Furthermore, most of the interventions sought to address gender norms around family planning use - mostly to promote male support for their partner’s use, but also some to promote male use of methods. The interventions found, as other cross sectional studies show, that men want information on family planning and the notion that family planning is women’s business only is no longer true. When male methods, notably condoms, vasectomy and SDM, were made available through interventions, uptake generally increased. Finally, a number of the interventions had positive outcomes related to promoting more equitable gender norms related to family planning and increasing couple communication on fertility and contraceptive use.

### Key considerations in programming for men as contraceptive users

The review of programming, in addition to the broader literature on gender and family planning and interviews with experts, identified a number of issues that are important to consider in programming for men as family planning users (Table 2).

**Table 2:** 10 Key considerations in programming for men as family planning users

• Provide information and services to men and boys where and when they need it • Address gender norms that affect men’s use of contraceptive methods • Improve couple and community communication • Meet men’s needs while respecting women’s autonomy • Link men’s family planning use with their desire to support their families • Teach adolescent boys about pregnancy prevention and healthy sexual relationships • Develop national policies and guidelines that include men as family planning users • Scale up programs for men • Fill the gaps through monitoring, evaluation, and implementation science • Create more contraceptive options for men	

#### Provide contraceptive information and services to men and boys where and when they need it

Reproductive health services are largely seen as spaces for women, with male norms of masculinity often challenged by men’s accessing reproductive health services [11–13]. Most family planning services are perceived as not welcoming to men as either partners or clients in and of themselves, with a lack of health infrastructure targeting men, including policies, services and hours of services [14, 15]. Furthermore, the capacity of health workers to counsel men on contraception is lacking [16].

Programs could make better use of existing facilities by extending hours, or allocating time for men and couples [17]. Providing family planning through pharmacies and drug shops is a promising Family Planning High Impact Practice [18]. These venues may be preferred by men who are reluctant to attend health facilities, particularly if they have to travel long distances [19].

There is a large gap in men and adolescent boys’ access to information and services, leading to a disparity between male and female knowledge of and access to contraceptives. As Piet-Pelon et al. [20] reflected “…. How is it that we woke up so recently to the fact that men are likely to be just as out of the loop on correct information as their partners?.... How had we expected them to participate when we neither shared information nor provided them with accessible services?” (p. 61).

Adolescent boys and men want information to inform their sexual lives, enhance their understanding of contraceptive options, and to protect themselves from HIV but have limited options on where to obtain this information. Studies show that men sometimes obtain their information from the media (including pornography sites) or from other male friends but this information may not always be accurate. Many men stress the importance of communication around sex and reproduction but do not have skills to do so [21]. Men want to know about how to combat infertility and sexual inadequacies [22] and they generally want male providers with whom they can discuss these sensitive issues [23]. Men also want guidance on how to use a condom effectively but may be embarrassed to ask. Boys, too, were found to want information on sex, impotency, infertility, and erectile dysfunction just like married men [24]. In some countries, service providers are unwilling to provide contraceptive information or methods to unmarried adolescent boys [24].

Further, parents in many countries focus on adolescent girls and pregnancy, as “when girls become pregnant the responsibility rests mostly on their parents and not the boys” [25]. Health care workers may share this view, and therefore do little to provide information or contraception to adolescent boys [25]. Yet young men have reported a strong desire for information about sex-related matters, both from their families and the health system.

Hotlines are demonstrating promise as platforms to reach men with information about contraceptives and available services, in addition to other health information men need. In the Democratic Republic of Congo (DRC), evaluation of use of a family planning hotline – Ligne Verte – found that although women were the primary target of the hotline, men represented 80% of the 20,000 calls per year, with men asking for information about contraception and where to obtain it [26]. The vast majority (94%) of calls to a hotline in India were from males, who asked for a range of information, including about erectile disfunction, early ejaculation, masturbation, HIV/AIDs, anxiety about penis length, pre-marriage anxiety and guidance; infertility, nocturnal emissions, gender orientation; contraception, STI, and on consummation of marriage, among other topics [27]. Similarly, mHealth programming is increasing, with good participation from men [28–31].

Integrating contraceptive information in other development projects can take advantage of reaching men where they work. Interventions in El Salvador and Honduras successfully increased men’s knowledge and reported family planning use by incorporating information and referrals into agricultural work through volunteers talking to men in the fields while they were working [32].

#### Address gender norms that can limit or promote men’s participation in contraceptive use

Most programming is based on the perception that gender norms value masculinities that include bragging about sexual encounters, having frequent sex and multiple partners and leaving the responsibility for unintended pregnancies to women [33]. This view of gender norms surrounding sexuality, reproductive health and family planning reinforce the focus on female use of contraception. Gender inequitable norms can diminish the likelihood of discussions of contraception and joint decision-making on reproductive health [34]. Where gender inequality is strong, men are less likely to use a condom [35].

Masculine ideals may also involve showing respect for and protecting their female partners. Both views of masculinity can affect family planning use by women and men [36, 37]. Where men and boys have been exposed to gender equality programming, they are more likely to report increased contraceptive use, including condom use.

Equitable gender norms can positively impact use of vasectomy and that countries with current high rates of vasectomy are also ones that are more gender equitable (Personal communication with F. Verani, October 9, 2015). In Costa Rica, men who held more gender equitable (less “machista”) views pursued getting a vasectomy as part of their quest for emotional commitment to their wives and to the well-being of their wives. Men who got vasectomies saw themselves as being responsible for averting unintended pregnancies [38]. Vasectomies have increased in Costa Rica 76% from 2000 to 2003 and another 70% between 2003 and 2006, with low fertility linked to being a “modern” man. The method became so popular that there was a reported 2-years wait to get a vasectomy in a social security hospital in 2009.

Data from 58 Demographic and Health Survey (DHS) men’s surveys in 18 countries showed that men generally consider family planning their business as well as women’s [39]. In surveys in 12 countries, men were asked whether they agreed with the statement, “contraception is a woman’s business and a man should not have to worry about it.” In all of the 12 countries included in the analysis (ten in Africa and two in Asia), over half of the men disagreed with the statement, and in six of the 12 countries, over 70% of the men disagreed.

Men’s views of gender equality and their roles in contraceptive decisionmaking are not monolithic. Severy [40] notes the importance of understanding the variation in power dynamics, both across and within cultures and relationships. Ethnographic work in Egypt illustrates that men do express concern for the health and wellbeing of their wives, at the same time they express their authority and power [41]. Indeed, many men considered their wives as partners, although they also recognized their socially mandated role as providers and felt a strong sense of responsibility for their families. Further, data from South Africa, Kenya and Uganda (1999–2000) indicated that men were no more likely than women to be against family planning [42].

Understanding the views of policymakers and program managers on men’s use of contraception is also important since that has implications for policy development and program implementation. For example, in Benin, Diakite [43] notes that one barrier to male engagement in family planning, including as users, is “lack of interest and appreciation for men in government FP initiatives.” Writing about Indonesia and citing statistics on use of condoms, vasectomy and withdrawal, which ranged from 3.1% in 1987 to 2.8% in 2003, Hull [44] claims that Indonesia missed an opportunity to promote men’s use of contraception. He explained that:

“Leaders in the community and the family planning program were remarkably conservative about the idea of promoting male methods. Increasingly, they questioned the efficacy of condoms and the acceptability of vasectomy, opting to ignore the fact that the public was quite interested in trying male methods…. Indonesia saw a steady decline in reported condom use for family planning (and for HIV prevention) and the failure of relatively inexpensive male sterilization to reach even one-ninth of the number of female sterilizations” P. 245.

#### Improve couple and community communication

Couple communication is important for improving contraceptive use and gender equity and is a component of many interventions to promote male engagement [45, 46]. When couples have discussed family planning, contraceptive use tends to go up, with recent evidence from Kenya [47]; Ethiopia [48–50]; Nepal [51]; Bangladesh [52] and Pakistan [53, 54]. An analysis of DHS survey data from Bangladesh, for example, found that discussions between husband and wife on family planning was the single most significant effect on both current contraceptive use and modern method preference [52]. Promoting couple communication should include encouraging partners to discuss use of contraception by either partner.

Promoting dialogues among community members can also increase gender equality and thus affect men’s and women’s sexual and reproductive health, including contraception. Related programing examples include the GREAT Project in Uganda [55], Stepping Stones in South Africa [56, 57], SASA! in Uganda [58, 59], CARE’s Results Initiative in Kenya [60], and Program H in Brazil [61, 62]. These program examples show that when informed by the need to address inequitable gender norms, increasing community dialogues and couple communication about power dynamics, the need for gender equality and the importance of contraception may lead to a number of positive public health and gender equity outcomes.

Some have noted the need to ensure that couples communication interventions take into account unequal power in relationships [63] and Green ME, Barker G, Olukoya A, Pawlak P, Contreras JM and Heilman B: What happens when we engage men?, unpublished. CARE’s Family Planning Results Initiative, implemented in western Kenya from 2009 to 2012, found that while both women and men described a shift towards gender equitable roles, “many couples still reported that men were the primary decision-makers within relationships” (P. 7) [60]. While women in the project intervention described many benefits of partner use of and support for family planning, for those women who did face opposition from their partners, the women “specifically discussed the critical importance of their ability to independently access family planning” (P. 40) [60].

Shifting power dynamics in couple communication can involve incrementally subtle shifts. In Malawi, an intervention using male motivators evaluated through a randomized design with 400 men divided between the intervention and control groups, found that contraceptive use increased significantly in the intervention group (78%) compared to the control group (59%), with men’s discussions with their wives as a significant factor in family planning uptake [64]. Of the men who reported family planning uptake, 56% reported using condoms. 1 year following the intervention, 30 female partners were also interviewed. As a result of the male motivator program, all men noted the importance of increased information and knowledge in contributing to spousal communication and family planning use, with the majority of men and women reporting more frequent and easier communication between spouses, particularly in relationship to family planning [64]. The women also appreciated the male motivator program for reaching their husbands and addressing their opposition to family planning, making it easier for couples to discuss family planning.

The study in Malawi analyzed the gendered patterns of decisionmaking, finding that few participants said that it was the man’s role to decide on family planning. Yet, the findings also showed that a majority of women and men described the decision to use family planning as being made by men, while the decision of what method to use was more mixed between women, men and couples together. While this appears to strengthen men’s domination of women, the reality in Malawi was more subtle. The authors concluded:

“Despite that many women still played a passive role in communication, that they are consulted at all represents a shift and was described as such by the women. This is also supported by the increased respect women felt they received from their partners and by perceived improvements in their relationships as a result of improved communication. Although the ideal of equal decision-making may not yet be a reality, this represents a positive change in that direction” (P. 816) [64].

#### Meet men’s needs while respecting women’s autonomy

Although engaging men in family planning, as users and supportive partners, is gaining acceptance, there is concern about effects of male engagement on women’s autonomy and the fundamental human right for women to control “their own sexuality, their bodies and their health” (P. 10) [65]. Some women may not want men to be responsible for contraception, fearing that a mistake or omission by their partner may lead to an unintended pregnancy [22]. It is critical, then, to learn from early mistakes made in programming to promote male engagement that appealed to stereotypic gender norms [66, 67], and to make sure that interventions to encourage male contraceptive use do not disempower women and reinforce gender inequalities [34]. The United Nations Population Fund (UNFPA), which supports husband’s schools to reach men and influence them to support women’s reproductive health, reports that couple communication has increased and men have a greater understanding of the importance of the health and wellbeing of their wives and children [68]. However, an evaluation of the husbands’ schools by the Institute for Reproductive Health will help show if the approach promotes gender equity or reinforces male dominance.

Men should be able to exercise their reproductive right to use contraception, but they should not be able to deny their partner’s right to use family planning. While highlighting the benefits of involving both men and women in contraception, women must retain the ability to choose to use contraception and to access services alone (Stern, [69]; Tao et al. [70]).

#### Link men’s family planning use with their desire to support their families

More attention has recently been focused on men as engaged fathers [22]. Whether an adolescent boy or a man wants to become a father is an important question with potential consequences not just for the women or adolescent girl who becomes pregnant but also for the man or adolescent boy. Men’s attitudes have been changing and men want to be involved in decisions about having or not having children [71]. A 2014 study in Pakistan found that one of the main motivators behind a trend of wanting smaller families is economic stress and pressure men feel about being the main income earners for their family [72]. Another study of married men in Pakistan found that the perception that a responsible, caring husband uses family planning to improve the standard of living of his family and to protect his wife’s health made men 1.5 times more likely to intend to use a condom [73].

In Jamaica, the Women’s Center Foundation has a longstanding and successful program to help school-age girls who get pregnant stay in school and delay having more children [74]. A study in 2002 of the “baby fathers” of girls who had been in the program between 1990 and 1994 [75], found that the boys considered themselves better fathers than their fathers had been but “many felt that a separate program for men could help them communicate with their sons about reproductive health, sexuality, and personal development” (P. 20) [2].

#### Teach adolescent boys about pregnancy prevention and healthy sexual relationships

Sexuality education that is gender transformative is more effective in pregnancy prevention than sexuality education that does not challenge gender norms [76]. Comprehensive sexuality education therefore “must talk about parenthood and make the link between sexuality and fatherhood more explicit” (P. 139) [21]. Many adolescent pregnancy prevention programs reinforce gender inequality by reinforcing the notion that girls are the only ones responsible for preventing an unintended pregnancy [12]. Almost nothing is known about the impact of early, unintended and unwanted pregnancy on the lives of adolescent boys [77]. Some experts have recommended the need for age appropriate educational programs on sexual and reproductive health, including how boys can responsibly prevent unintended pregnancy [16] and Todesco M and Gay J: Guidance for the education sector on preventing unintended adolescent pregnancies, forthcoming from UNESCO.

#### Develop national policies and guidelines that include men as family planning users

Ensuring that constructive male engagement, including men as users, is included in national policies and guidelines is nascent [4, 78]. A review of public policies to promote gender equality in Mexico, South Africa, Chile, India and Brazil found gaps in health policies, among other sectoral policies, and noted the need for policies that both engage men in supporting their partners in contraceptive use but also that promote men’s use of male methods [79].

Some countries have successfully integrated policies regarding men’s involvement in sexual and reproductive health. Cambodia was among the first countries to do so in 2003. These guidelines, including reaching men as family planning users in addition to being supportive partners, were integrated into the 2006–2010 National Strategic Framework for Reproductive and Sexual Health in Cambodia [80].

A number of countries have developed Family Planning Costed Implementation Plans (CIPs) where family planning activities are planned and costed with a view to scaling up programming. Reviewing a country’s CIP for male inclusion is one concrete way to ensure adequate focus on men in family planning programming. Analysis of five CIPs from African countries (Ghana, Malawi, Uganda, Senegal, and Tanzania) suggests that most programming for men focuses on increasing their support for their partners’ use of contraception with little attention on increasing men’a own use of contraception [81–85].

#### Scale up programs for men

Programming for men as contraceptive users is scant, although growing, and even interventions to engage men in family planning as partners have remained small scale and short term [69, 86–88]. There are, however, some approaches that have demonstrated success in constructive male engagement, including as contraceptive users, and could be scaled up at regional and national levels. Multidimensional programs have the potential to show even greater impact if carried out with a wider approach over a longer term.

Communications programming in Bangladesh, Ghana, Honduras, Guatemala, Pakistan, India, Vietnam, Burkina Faso, Tanzania, and Nicaragua show that mass media can create national conversations that address the social and cultural barriers to men’s contraceptive use. Social media is a rising force that can be harnessed to connect men to information and services as seen in the use of Facebook by WINGS in Guatemala to publicise the availability of vasectomy [89]. Social marketing can challenge social norms and help overcome barriers to acceptability of contraceptive use. Use of male motivators and other trained male peers has repeatedly demonstrated success on a small scale. This programming could be more effective on a large scale if it were linked with other strategies, such as comprehensive sexuality education that includes information on men and boys’ contraceptive needs; provider training and information and service provision for men; and gender-responsive programming that improves couple communication and joint decisionmaking. The FALAH Project, implemented between 2008 and 2012 in Pakistan shows that programming for men can reach millions of men. That project spanned 20 districts across four provinces and reached more than 9 million women and men [54]. The FALAH Project engaged men through multiple strategies including male motivators and peers, mass media, theatre performances, men’s groups, and religious leaders. An mHealth solution in India that supports fertility awareness and use of SDM (CycleTel Family Advice and CycleTel Humsafer) has been developed by the Institute for Reproductive Health in partnership with a private sector organization [30]. Its ongoing implementation will provide information on managing a commercial partnership as a pathway to scale and sustainability.

An ongoing challenge will be ensuring enough supply of vasectomy services and condoms to meet the growing demand that would be generated through scaled up programming for men. Experience in KwaZulu Natal in Western Cape, South Africa, shows that with political will, this challenge could be met: After a significant drop in condom use in South Africa at last sex between 2008 and 2012 [90], KwaZulu Natal officials distributed 58.9 condoms per adult male in 2014, up dramatically from 8.2 condoms per male in 2010 [90]. While this effort to increase condom use was based on HIV prevention rather than for increasing uptake of male contraceptive methods, it shows how political will can dramatically scale up condom distribution, facilitating greater availability for use.

#### Fill the gaps through monitoring, evaluation, and implementation science

Review of programming for men as family planning users found few robust evaluations of programs to engage men, let alone programs directed at men as contraceptive users. Many of the male-focused programs have positioned men as partners and supporters of women’s contraceptive use and thus it is difficult to separate those interventions from those aimed at increasing male contraceptive use and engaging men in their own sexual and reproductive health. Furthermore, not all programs report findings disaggregated by sex, and by contraceptive method, making it difficult to determine the effect of programming on male use of methods. Few studies ask men directly about their experiences with contraception, but instead are most likely to ask female partners what changes have occurred in male partners’ attitudes and practices based on interventions with women [23, 91–93].

If men are continually viewed as obstacles or accessories to women’s health, it will be impossible to meet men’s needs. A greater understanding of men’s contraceptive and reproductive health needs begins first with the recognition of their rights to information and services for them. More systematic data collection on men’s fertility and relationships could greatly enhance information and service provision for men. Beyond that, there is a need to better understand the process of change for men, rather than just providing outcomes [69]. There is little literature on factors to increase uptake or barriers that prevent men from using contraception from the viewpoint of men themselves [34]. Critical research is missing on what are effective programs to increase responsibility by adolescent boys, prior to sexual activity, to prevent unintended pregnancy through male or female contraceptive use, as well as to ensure dialogue on pregnancy prevention, and that the sexual act is consensual.

Regional and country contexts are important in developing this research.

Funding for research and evaluation remains an ongoing challenge. Some efforts would benefit from rigorous evaluation but do not have funds to carry out endline surveys or evaluate that data. For example, there is a current effort to increase contraceptive use among both boys and girls in Togo using the “It’s All One” curriculum, but evaluation of gender-related and contraceptive uptake outcomes is needed (Personal communication with M. Toliver, October 23, 2015). The curriculum *Gender Matters, or Gen.M*, developed in the United States, [94] promotes gender equality and teaches boys about their roles and responsibilities in preventing unintended pregnancies, including that they can use contraception, can be adapted for use in developing country settings but such adaptations would need to be evaluated (Personal communication with J. DeAtley, December 2, 2015).

Programming to promote existing male methods could benefit from further evaluation. For example, though it does not have recurrent costs, SDM requires counseling and provider training. Further evaluation of SDM programming could lead to a greater understanding of how to better position the method for increased use. Condom programming would benefit from additional research on how to sustain condom use after marriage, especially as a dual strategy. Vasectomy programming may benefit from further evaluation of successful interventions that have dispelled myths and fears of loss of potency for men and that have changed community norms around acceptance of male sterilization. While programs do not promote withdrawal, research could help position it at least as a method couples could use if needed in the absence of another method.

#### Create more contraceptive options for men

Review of programming for men reinforces that men need additional contraceptive options beyond the methods currently available to them. There is sizable demand for a novel male contraceptive option, particularly for reversible contraception [34, 95]. In a survey of more than 9,000 men ages 18–59 across nine countries, 28.5–71.4% of men of various nationalities expressed a willingness to use a hormonal male contraceptive [96]. Dorman and Bishai [97] note that “even assuming that only 25% of men who indicated they would ‘definitely’ or ‘probably’ use male contraception in the Heinemann et al. survey, the estimated number of potential users ages 15–64 years in those nine countries alone is close to 44 million” (p. 5) [98]. Surveys of women’s acceptability of a male pill in Edinburgh, Cape Town, Shanghai and Hong Kong have shown that while women may not trust men in general, they were more trusting when it came to their own partner [95, 98]. A study in south-west Nigeria of 329 men ages 18 – 60 years who were married or cohabiting and had at least one child found that most men would be willing to accept reversible male contraception [99]. Shand found in field interviews in Malawi that men stated that the best family planning methods were for women and therefore, men do not use contraception (Personal communication with T. Shand, October 15, 2015).

Work to develop additional male methods of contraception has been going on for decades with a focus on hormonal and non-hormonal approaches. Creation of novel hormonal methods for men has stalled, however. Many of the attempted hormonal formulations created for men have had a number of intolerable side effects [97]. A recent trial of a hormonal contraceptive injection for men, which was found to be nearly as effective as the pill for women, was halted early due to side effects [100]. In spite of the side effects, three-quarters of the men in the trial said they would be willing to continue to use the method if it were available [101]. Reformulation of the hormones may decrease the side effects. Currently, some promising new non-hormonal methods for men are under development, including Vasalgel, the RISUG method, and Gendarussa [102].

Funding is a challenge for male contraceptive development. Pharmaceutical companies are not currently investing in new contraceptive development, leaving smaller efforts by non-profit organizations and foundations to fill the gaps. With adequate funding, there could be a new male contraceptive on the market within a decade. Without adequate funding, efforts may take 20 or more years to bear fruit.

## Conclusion

Review of programming for men as family planning users shows that currently, men and boys are not particularly well served by programs. Most programs operate from the perspective that women are contraceptive users and that men should support their partners, with insufficient attention to reaching men as family planning users in their own right. At the same time, the review highlighted that there is sufficient evidence demonstrating men’s desire for information and services, as well as men’s positive response to existing programming to warrant further programming for men and boys in family planning and contraceptive services. Review of programming shows strategies for reaching men with evidence ranging from proven (social marketing; and outreach with male motivators and peer educators/menotors), to promising (clinic provision of information and services; mass media; promoting community dialogue and comprehensive sexuality education), and emerging (social media; mHealth; hotlines; and engaging religious leaders). These strategies, along with recommendations for further implementation science highlights the need to strengthen programming to engage men as family planning users in addition to efforts to address gender-based norms and behavior that hinder contraceptive use. This paper also articulates key considerations that should be taken into account to orient programming to reach men and boys with the information and services they need to be contraceptive users. While programming for men should not compromise women’s autonomy, it should also not be implemented with the assumption that contraceptive use is only for women.

Scaling up successful programming and ensuring that the key considerations for programming are intregated into interventions for men, and continuing to undertake robust implementation research and valuation, will increase men’s knowledge and use of family planning services, reduce barriers, increase gender equality and improve the health and well being of men and women, boys and girls worldwide.

## Abbreviations

CIP: Costed Implementation Plan
DHS: Demographic and health surveys
FALAH: Family advancement for life and health project
GREAT: Gender roles, equality, and transformation project
ICPD: International Conference on Population and Development
SDM: Standard Days Method
UNFPA: United Nations Population Fund
